# Parkinsonian Syndrome with Frontal Lobe Involvement and Anti-Glycine Receptor Antibodies

**DOI:** 10.3390/brainsci10060399

**Published:** 2020-06-23

**Authors:** Dominique Endres, Harald Prüss, Michel Rijntjes, Tina Schweizer, Rita Werden, Kathrin Nickel, Sophie Meixensberger, Kimon Runge, Horst Urbach, Katharina Domschke, Philipp T. Meyer, Ludger Tebartz van Elst

**Affiliations:** 1 Section for Experimental Neuropsychiatry, Department of Psychiatry and Psychotherapy, Medical Center-University of Freiburg, Faculty of Medicine, University of Freiburg, 79104 Freiburg, Germany; tina.schweizer@uniklinik-freiburg.de (T.S.); rita.werden@uniklinik-freiburg.de (R.W.); kathrin.nickel@uniklinik-freiburg.de (K.N.); sophie.marie.meixensberger@uniklinik-freiburg.de (S.M.); kimon.runge@uniklinik-freiburg.de (K.R.); tebartzvanelst@uniklinik-freiburg.de (L.T.v.E.); 2 Department of Psychiatry and Psychotherapy, Medical Center-University of Freiburg, Faculty of Medicine, University of Freiburg, 79104 Freiburg, Germany; katharina.domschke@uniklinik-freiburg.de; 3 Department of Neurology and Experimental Neurology, Charité-Universitätsmedizin Berlin, 10117 Berlin, Germany; harald.pruess@charite.de; 4 German Center for Neurodegenerative Diseases (DZNE) Berlin, 10117 Berlin, Germany; 5 Department of Neurology, Medical Center-University of Freiburg, Faculty of Medicine, University of Freiburg, 79106 Freiburg, Germany; michel.rijntjes@uniklinik-freiburg.de; 6 Department of Neuroradiology, Medical Center-University of Freiburg, Faculty of Medicine, University of Freiburg, 79106 Freiburg, Germany; horst.urbach@uniklinik-freiburg.de; 7 Center for Basics in Neuromodulation, Faculty of Medicine, University of Freiburg, 79106 Freiburg, Germany; 8 Department of Nuclear Medicine, Medical Center-University of Freiburg, Faculty of Medicine, University of Freiburg, 79106 Freiburg, Germany; philipp.meyer@uniklinik-freiburg.de

**Keywords:** parkinsonian syndromes, frontal dementia, glycine receptor, antibody, PERM, stiff-person syndrome

## Abstract

*Background:* Atypical Parkinsonian syndromes with prominent frontal lobe involvement can occur in the 4R-taupathies progressive supranuclear palsy (PSP) and corticobasal degeneration (CBD). Secondary forms of movement disorders may occur in the context of autoimmune encephalitis with antineuronal antibodies, such as anti-glycine receptor (anti-GlyR) antibodies, which are typically associated with Stiff-Person spectrum syndrome, or progressive encephalomyelitis with rigidity and myoclonus. Overlaps between neurodegenerative and immunological mechanisms have been recently suggested in anti-IgLON5 disease. In this case study, the authors describe a patient with a Parkinsonian syndrome with frontal lobe involvement and anti-GlyR antibodies. *Case presentation*: The patient presented was a 63-year-old female. Her symptoms had begun with insomnia at the age of 60, after which, since the age of 61, increasing personality changes developed, leading to a diagnosis of depression with delusional symptoms. Severe cognitive deficits emerged, along with a left-side accentuated Parkinsonian syndrome with postural instability. The personality changes involved frontal systems. Magnetic resonance imaging (MRI) showed low-grade mesencephalon atrophy. [^18^F]fluorodeoxyglucose positron emission tomography (FDG PET) depicted a moderate hypometabolism bilateral frontal and of the midbrain, while [^123^I]FPCIT single-photon emission computed tomography (SPECT) revealed severely reduced dopamine transporter availability in both striata, indicating pronounced nigrostriatal degeneration. In addition, anti-GlyR antibodies were repeatedly found in the serum of the patient (max. titer of 1:640, reference: <1:20). Therefore, an anti-inflammatory treatment with steroids and azathioprine was administered; this resulted in a decrease of antibody titers (to 1:80) but no detectable clinical improvement. The cerebrospinal fluid (CSF) and electroencephalography diagnostics showed inconspicuous findings, and negative CSF anti-GlyR antibody results. *Conclusion*: The patient presented here was suffering from a complex Parkinsonian syndrome with frontal lobe involvement. Because of the high anti-GlyR antibody titers, the presence of an autoimmune cause of the disorder was discussed. However, since no typical signs of autoimmune anti-GlyR antibody syndrome (e.g., hyperexcitability, anti-GlyR antibodies in CSF, or other inflammatory CSF changes) were detected, the possibility that the anti-GlyR antibodies might have been an unrelated bystander should be considered. Alternatively, the anti-GlyR antibodies might have developed secondarily to neurodegeneration (most likely a 4-repeat tauopathy, PSP or CBD) without exerting overt clinical effects, as in cases of anti-IgLON5 encephalopathy. In this case, such antibodies might also potentially modify the clinical course of classical movement disorders. Further research on the role of antineuronal antibodies in Parkinsonian syndromes is needed.

## 1. Background

Parkinson’s disease is characterized by rigidity, tremor, shuffling gait, and other symptoms, such as loss of smell [1]. Atypical Parkinsonian syndromes include progressive supranuclear palsy (PSP), corticobasal degeneration (CBD) and multisystem atrophy.

Different PSP phenotypes include classic Richardson’s syndrome, PSP-Parkinson’s syndrome, PSP-corticobasal syndromes, PSP-speech language syndrome, PSP with predominant cerebellar ataxia, and PSP with frontal presentation [2]. Richardson’s syndrome is characterized by mostly symmetrical, axially accentuated, akinetic-rigid Parkinson’s syndrome, as well as a vertical supranuclear gaze paresis, early falls, and a poor response to levodopa [2,3]. PSP with frontal presentation is rare, often showing the symptomatology of a behavioral variant of frontotemporal dementia years before the motor features of PSP become evident [2,4]. The pathological hallmarks are microtubule-associated tau proteins in neurofibrillary tangles (so-called 4-repeat tauopathy), oligodendrocyte coils, and astrocytic tufts [5]. Magnetic resonance imaging (MRI) characteristically shows atrophy in the midbrain and superior cerebellar peduncles, while [^18^F]fluorodeoxyglucose positron emission tomography (FDG PET) displays hypometabolism of the frontal cortex, caudate, midbrain and thalamus [2], of which the midbrain findings have been incorporated in the novel diagnostic criteria as a supporting imaging finding [5]. Dopamine transporter (DAT) imaging [e.g., [^123^I]FPCIT single-photon computed tomography (FPCIT SPECT)] demonstrates severely and fairly symmetrically reduced nigrostriatal innervation [6]; cerebrospinal fluid (CSF) tau/p-tau levels are normal or even lower than those of controls [2]. PSP has a poor prognosis because no causal or sustained effective symptomatic treatment is currently available [2,3].

The four most important CBD subtypes are probable corticobasal syndrome (CBS), frontal behavioral-spatial syndrome, nonfluent/agrammatic variant of primary progressive aphasia, and PSP syndrome [7]. CBS is characterized by asymmetric akinetic-rigid Parkinsonism, limb dystonia or limb myoclonus, and other cortical symptoms, such as ideomotor apraxia or alien limb phenomena [8]. Like PSP, CBD is also a 4-repeat tauopathy [9]. MRI can show asymmetric frontoparietal atrophy in the course of the disease; the brain stem MRI findings are typically inconspicuous [3]. FPCIT SPECT reveals asymmetric reduction of the presynaptic dopamine transporter density, and FDG PET displays usually markedly asymmetrically reduced frontoparietal glucose metabolism [6,10,11]. There is also no known causal treatment [8]. 

Secondary forms of movement disorders may occur in the context of autoimmune encephalitis with different antineuronal antibodies [12,13,14,15]. Dyskinesias are found in anti-NMDA-receptor (R) encephalitis. Cerebellar ataxia is associated with a number of paraneoplastic, antineuronal antibodies against intracellular antigens. PSP phenotypes are described for anti-Ma2 and anti-IgLON5 antibodies, Stiff-Person spectrum diseases are associated with anti-GAD5 antibodies or anti-glycine receptor (anti-GlyR) antibodies, and progressive encephalomyelitis with rigidity and myoclonus (PERM) syndrome is associated with anti-GlyR antibodies [13,16]. 

Patients with anti-GlyR antibody syndrome are, on average, 50 years old and suffer from spasms, stiffness, rigidity, myoclonus, and related walking difficulties (with falls in 80%). Limb paresis and pyramidal signs are observed in 60% of cases, trigeminal, facial, and bulbar disturbance or excessive startle reflexes in 57%, and oculomotor disturbances with nerve or gaze palsy in 53%; cognitive impairment is present in half of patients [17]. A recent publication has pointed to an extension of the spectrum with several visual symptoms, including visual hallucinations, synesthesia and intermittent diplopia [18]. Subacute courses occur in 44% of cases, while a primary chronic course has been described in 11% [17]. Tumor association is observed in <5% [16]. MRI of the brain reveals pathological findings in 28% of patients, and spinal lesions in 22%. Electroencephalography (EEG) pathologies have been found in 71%, with slowing in about half (52%) [17]. CSF abnormalities are observed in about half of the cases [19], with increased white blood cell (WBC) counts in most (43%) [17]. Intrathecal anti-GlyR antibody synthesis was observed in the majority of cases [17,20,21]. The response to anti-inflammatory treatment is mostly good, with the modified Rankin scale scores dropping from a median of 5 during disease peak to 1 [17]. 

Overlaps between neurodegenerative and immunological mechanisms have been previously described in anti-IgLON5 disease [22,23]. In anti-IgLON5 encephalopathy, antibodies against the neuronal cell-adhesion protein IgLON5 have been shown to be associated with tauopathy of the brainstem tegmentum [22].

Rationale: The symptoms of Parkinsonian syndromes with frontal lobe involvement, such as PSP or CBD, and autoimmune anti-GlyR antibody syndrome have obvious overlaps. From a clinical perspective, the distinction between the two is important because of the different therapy options and prognoses indicated. New developments in the field also raise the question of the interaction between neurodegenerative and immunological diseases. The case presented here shows a patient in this conflicting area, with Parkinsonsian syndrome with frontal lobe involvement and anti-GlyR antibodies.

## 2. Case Presentation

The female patient was 63 years old when presented to our hospital. She has given her signed written informed consent for this case report, including all the data and the presented images, to be published. At that time, she suffered mainly from personality changes (reduced energy, social withdrawal, introversion, emotional flattening, avoidance of eye contact, loss of interest in discussions, lack of insight), cognitive deficits (attention/concentration and working memory deficits), and Parkinson’s syndrome with left-emphasized bradykinesia/reduced amplitude in finger tapping, hypomimia, bilateral rigor, and a postural instability with a tendency to fall (Hoehn and Yahr stage IIb).

**Figure 1:**
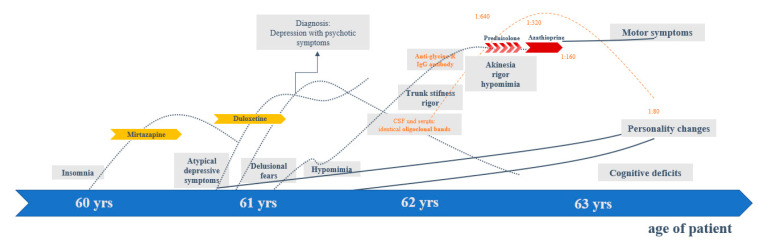
Clinical course. Abbreviation: CSF, cerebrospinal fluid; yrs, years.

Up to the age of 60, the patient had always been mentally healthy, in a good mood, and active. At the age of approximately 60 years, she developed growing insomnia (with problems in maintaining sleep and early awakening). At the age of 61, a change in her behavior became increasingly apparent. Her energy levels were reduced, she withdrew socially, seemed affectively flattened, and experienced more insecurity and anxiety. In addition, the patient developed delusional fears (felt spied on and therefore wanted the roller shutters closed continuously). Consequently, depression with psychotic symptoms was diagnosed. Muscle tone was assessed as normal at that time. However, retrospectively, a reduced facial expression had already been observed by her husband. The sleep disturbances improved with low-dose mirtazapine (3.75 mg/day). Treatment with duloxetine (60 mg/day) led to slight improvement of delusional fears. In the further course, an increasing personality change was observed. The patient was less happy, more introverted, appeared to be increasingly emotionless, and was no longer able to maintain eye contact. The MRI examination of the brain and the EEG remained inconspicuous (at the age of 61 years). In CSF diagnostics, identical oligoclonal bands (OCBs) in CSF and serum were found, indicating a systemic process. The neurodegeneration markers (tau, p-Tau, β-amyloid quotient, 14-3-3) were in the normal range. At the same time, she developed increasing trunk stiffness and left accentuated rigor. At the age of 62, elevated anti-GlyR IgG serum–antibodies with a titer of 1:640 (reference: <1:20; Laboratory Krone, Bad-Salzuflen, Germany) were found, and a left-accentuated Parkinson’s syndrome became more and more apparent. Anti-inflammatory treatment with prednisolone (3 × 1000 mg/day) was started and repeated five times every 4 weeks. Tolerability was good, but no relevant clinical improvement was observed. The antibody titers only changed by one titer step (to 1:320). Thus, maintenance therapy with azathioprine (150 mg) was started. On this therapeutic regimen, a stabilization of the symptoms could be achieved, but no sufficient improvement. The antibody titers dropped to 1:80 (reference: <1:20). Additional levodopa treatment (with only 187.5 mg/day) was well tolerated, and led to the slight improvement of mood and rigor (see Figure 1).

### 2.1. Diagnostic Findings

**Figure 2:**
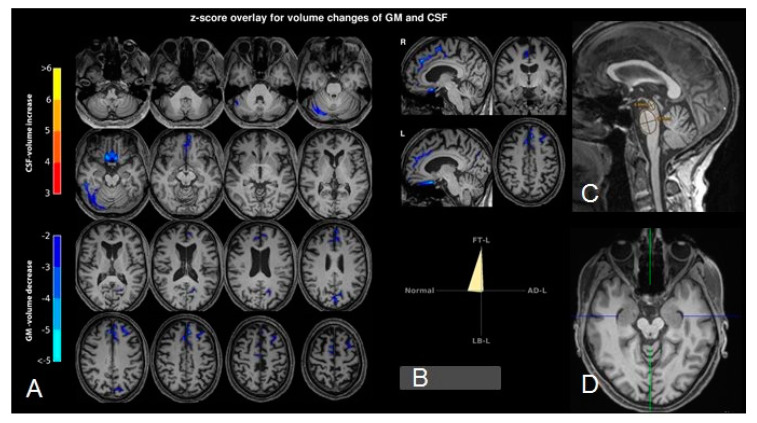
(**A**) Combined voxel- and region-based morphometry shows decreased gray matter volumes of the medial frontal lobes (z-scores < −2 are displayed in blue to white). (**B**) A support vector machine suggests this pattern to be compatible with a fronto-temporal lobar degeneration. On visual analysis, the midbrain is atrophic (**C**,**D**), however the midbrain to pons ratio is (with 0.61) in the normal range (**D**).

**Figure 3:**
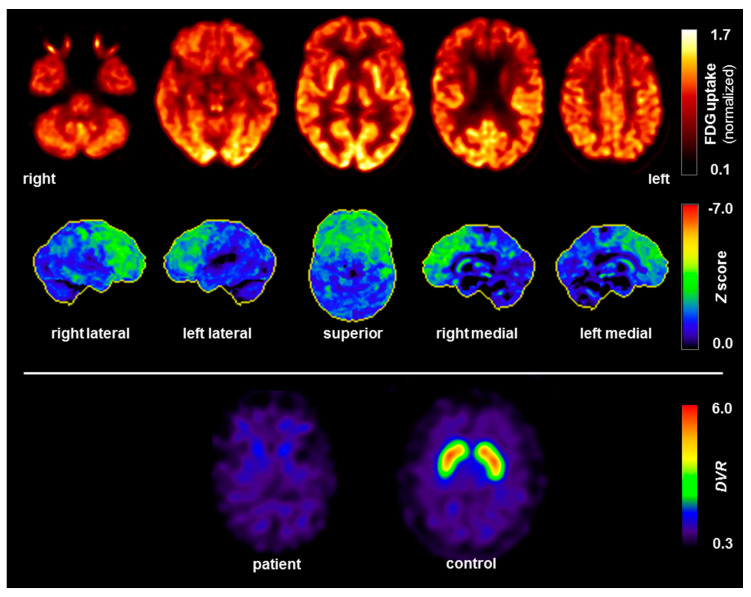
[^18^F]fluorodeoxyglucose positron emission tomography (FDG PET; upper panel) and [^123^I]FPCIT single-photon emission computed tomography (FPCIT SPECT; lower panel). The FDG PET revealed moderate bilateral hypometabolism of the frontal lobes, midbrain (see medial view), and, to a lesser extent, the caudate nuclei as well [upper row: transaxial FDG PET images, voxel-wise FDG uptake normalized to whole brain uptake; lower row: 3D surface projections of regions with decreased FDG uptake, color-coded Z-score compared to age-matched healthy controls [24]]. FDG PET of the brain was performed in addition to the whole-body scan at 50 min after injection of 348 MBq FDG (Vereos Digital PET/CT, Philips Healthcare, Eindhoven, The Netherlands). The FPCIT SPECT showed a severely reduced dopamine transporter (DAT) availability in both striata in the patient (left) compared to normal DAT binding in a healthy control (right), indicating pronounced nigrostriatal degeneration. FPCIT SPECT was performed 3 h after injection of 183 MBq FPCIT (Intevo SPECT/CT, Siemens, Erlangen, Germany; shown are parametric maps of the distribution volume ration (*DVR*) using the occipital cortex as reference without DAT binding). Abbreviations: R, right; l, left.

**Figure 4:**
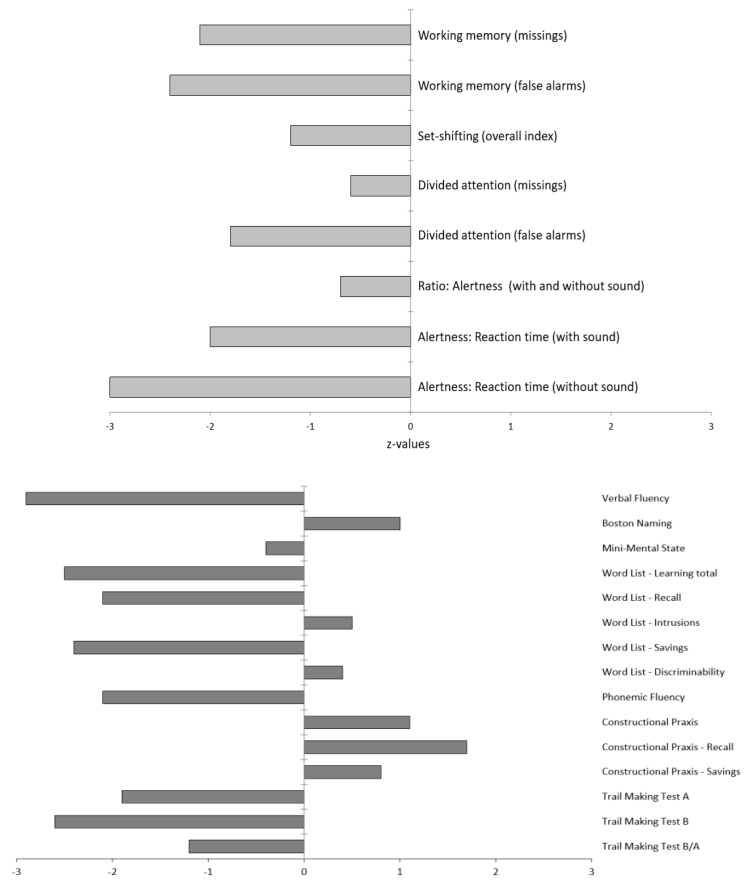
Neuropsychological test results. The testing of attentional performance (TAP) is shown above, and the “Consortium to Establish a Registry for Alzheimer’s Disease” (CERAD) test battery findings below. Z-values lower than 1 are below average, above 1 above average.

Following admission to our specialized ward at the age of 63, three years after the onset of the neuropsychiatric syndrome, a comprehensive clinical examination was performed. The MRI of the brain revealed low-grade mesencephalon atrophy, but there was no clear “hummingbird sign”. Other brain regions, including the basal ganglia and limbic system, were unremarkable (Figure 2). The MRI of the spinal cord showed no evidence of myelopathy or contrast-enhancing lesions. In the visual assessment and independent component analyses, the EEG was normal. The CSF revealed slightly increased protein concentration. The anti-GlyR serum IgG antibody titers were 1:80 (reference: <1:20; Laboratory Krone, Bad-Salzuflen, Germany), and the CSF testing was negative (identical OCBs in serum and CSF were no longer positive). Tau, p-tau and β-amyloid quotients were in the normal range. In addition, slightly elevated anti-thyroid peroxidase (TPO) antibodies were found in the serum (Table 1). The FDG PET depicted a moderate bilateral hypometabolism of the frontal lobes, the midbrain, and, to a lesser extent, the caudate nuclei as well, compatible with a frontotemporal lobar degeneration (Figure 3). On the whole body FDG PET/CT, there was no indication of a neoplastic process. For examination of nigrostriatal degeneration, which in severe expression would be more typical for PSP than FTD, an FPCIT SPECT was performed, which revealed a severely reduced dopamine transporter availability in both striates, indicating pronounced nigrostriatal degeneration (Figure 3). Thus, nuclear medicine findings are well compatible with PSP [5,6,11], whereas the lack of asymmetry argues against CBD [6,10,11]. In the neuropsychological testing of attention performance (TAP), a clear impairment of both basal and more complex attention functions was detected. The “Consortium to Establish a Registry for Alzheimer’s Disease” (CERAD) test battery revealed deficits in verbal memory and executive performance (word fluency, set-shifting; Figure 4).

### 2.2. Illness, Somatic, and Family History 

Her history was negative for in-utero/birth complications, febrile convulsions, inflammatory brain diseases, severe systemic infections, or craniocerebral traumata. She had no history of a neurodevelopmental or personality disorder. She never had a tumor disease but suffered from Hashimoto’s thyroiditis supplemented with l-thyroxin (75 µg/ day). Her father died early of a heart attack. Parents, grandparents, and siblings are not aware of any neurological, autoimmune or tumor diseases.

## 3. Discussion

This case study is of a female patient exhibiting a remarkable neuropsychiatric syndrome, beginning with insomnia and followed by increasing frontal lobe-linked personality changes. These changes initially mimicked depression with psychotic symptoms, and the patient later developed severe cognitive deficits and left-side accentuated Parkinson’s syndrome with postural instability.

In the absence of oculomotor abnormalities, the presence of a frontal syndrome and mild postural instability leads diagnosis towards a “suggestive frontal PSP” [5]. The (only) minimal improvement with levodopa and the tendency to fall would also be compatible with PSP [3]. A supranuclear palsy of the eye was not currently present, but this symptom can occur after a delay [2]. Alternatively, the clinical diagnosis could be probable CBD, with frontal behavioral-spatial syndrome and asymmetrical Parkinsonism [7]. The nuclear medicine findings, which showed hypometabolism of the frontal lobes, midbrain and caudate nucleus on FDG PET, as well as strongly reduced dopamine transporter availability in both striates on FPCIT SPECT, are well in line with PSP [5,6], whereas the lack of asymmetry in both examinations argues against CBD [6,10,11]. There was a low-grade midbrain atrophy, which did not decisively argue for or against one of the two 4-repeat tauopathies [5,25,26]. In summary, the patient appeared to be suffering from a Parkinsonian syndrome with prominent frontal lobe involvement, but a definitive PSP or CBD diagnosis could not be made. This is in line with the notion that the 4-repeat tauopathies PSP and CBD may be considered to represent different manifestations of a disease spectrum with several common clinical, neuroimaging, pathological, genetic and biochemical features, which prohibits accurate in vivo distinction between both diseases [11,27]. 

The elevated anti-GlyR antibodies (with a maximum titer of 1:640) suggested a possible autoimmune cause for the complex Parkinsonian syndrome with frontal lobe involvement. Initial positive identical OCBs in serum and CSF indicate a systematic process. The perinuclear signal in many neurons (likely reflecting ANAs) in the tissue-based assays could be indicative of increased autoimmune susceptibility. Anti-GlyR antibodies are typically associated with Stiff-Person spectrum syndromes or PERM, but they can also be syndromally associated with a very “colorful picture” of symptoms [17,21,28]. The presence of anti-GlyR antibodies in other neuro-immunological diseases, and in the “colorful clinical picture”, mitigates their high specificity, although these antibodies can be pathophysiologically relevant if they reach the brain [29]. The coexistence of Parkinsonian syndrome with frontal lobe involvement and anti-GlyR antibodies may relate to at least three possible causes and consequences:

1. The anti-GlyR antibodies could have been generated first, thereby triggering a clinical phenotype as a Parkinsonian syndrome with frontal lobe involvement. This is conceivable in principle, but very unlikely in the presented patient; no typical signs of autoimmune anti-GlyR antibody syndrome presently known (e.g., hyperexcitability, anti-GlyR antibodies in CSF, or other inflammatory CSF changes) were detected [17]. Furthermore, the instrument-based diagnostics, using EEG, MRI and FDG PET, as well as the poor response to anti-inflammatory treatment did not support the hypothesis of underlying autoimmune encephalitis.
2. The joint presence of a Parkinsonian syndrome with frontal lobe involvement and anti-GlyR antibodies could have been a pure coincidence. In this case, the anti-GlyR antibodies might be of no relevance. From the authors’ perspective, according to current knowledge, this alternative is possible. In antibody prevalence studies of healthy individuals, anti-GlyR antibodies have also been found in the serum of 0.06% of healthy individuals [one subject in a healthy control group of 1703 individuals [30].
3. Finally, a Parkinsonian syndrome with secondary anti-GlyR antibody production is plausible. In this case, the anti-GlyR antibodies could even have effects on the brain, but these would likely be overlooked in the progressive neurodegenerative Parkinsonian syndrome. In the authors’ view, this seems a plausible scenario. Indeed, a similar discussion is currently taking place regarding anti-IgLON5 antibodies; in patients with increased levels of anti-IgLON5 antibodies, a tauopathy of the brainstem tegmentum has been described in combination with antibody-mediated encephalopathy that is responsive to immunotherapy [22,23]. According to these novel observations, a new basic principle of interaction between neurodegeneration and neuroimmunological processes is conceivable. In the present case, it would be similar to encephalopathy with IgLON5 antibodies; antineuronal antibodies possibly modulate the clinical course of PSP/CBD or other neurodegenerative disorders, without primarily causing those disorders.

## 4. Conclusions

This paper describes a neuropsychiatric syndrome that initially manifested with purely psychiatric symptoms, and later developed into a Parkinsonian syndrome with frontal lobe involvement, likely caused by a 4-repeat tauopathy (PSP or CBD). A primary causal role of the additionally detected anti-GlyR antibodies was unlikely. However, the anti-GlyR antibodies might have developed secondarily to neurodegeneration, without overt clinical effects. Therefore, such antibodies might have the potential to modify the clinical course of classical movement disorders. Further research is needed on the role of immunological processes in neurodegenerative diseases like Parkinsonian syndromes.

## Figures and Tables

**Table 1:** Diagnostic findings approximately three years after symptom onset. Abnormal findings are marked in bold.

**Blood analyses**	Blood cell count, liver/kidney/pancreas values, and C-reactive protein were normal. Sodium and potassium were normal, **calcium levels were borderline high** (from 2.54–2.57 mmol/l; reference 2.15–2.5 mmol/l). Normal HbA1c. Folic acid and Vitamin B12 levels were normal. **Selenium (48 µg/l; reference: 75–140 µg/l) and Vitamin D (15 ng/mL; optimal: >30 ng/mL) were reduced.** Thyroid-stimulating hormone, triiodothyronine, and thyroxine levels were in normal ranges. **Autoantibodies against and thyroid peroxidase were increased (48.1 IU/mL; reference < 34 IU/mL).** Autoantibodies against thyroglobulin and TSH receptor were not increased. Antibody testing for Lyme borreliosis, syphilis, and HIV were negative. **Toxoplasmosis IgG antibodies were positive**, for IgM were negative. The Bartonella henselae and Hepatisis B/C/E serologies were negative. A quantiferon test was negative. No IgG autoantibodies against the following intracellular onconeural antigens, Yo, Hu, CV2/CRMP5, Ri, Ma1/2, SOX1, Tr, Zic4, or the intracellular synaptic antigens GAD65/amphiphysin, were found using Ravo line assay^®^. No IgG autoantibodies against the following intracellular onconeural antigens, amphiphysin, CV2/CRMP5, Ma2/Ta, Ri, Yo, Hu, recoverin, Sox1, Titin, Zic4, DNER/Tr, were found using immune blot method (Laboratory Krone, Bad Salzuflen, Germany). **In the serum IgG anti-GlyR autoantibodies with a titer of 1:80 (reference < 1:20) were discovered using cell-based assays (Laboratory Krone, Bad Salzuflen, Germany).** IgG autoantibodies against different other neuronal cell surface antigens (NMDA-R, AMPA-1/2-R, GABA_B_-R, DPPX, CASPR2, LGI1) were negative (using fixed cell biochip assays from Euroimmun^®^). No antibodies against IgLON5 or mGluR5/1 were detected using cell-based assays (Laboratory Krone, Bad Salzuflen, Germany). Aquaporin 4 and MOG antibodies were negative. **“Tissue-based assay” using indirect immunofluorescence on unfixed murine brain tissue (Prof. Prüss, Berlin) showed a perinuclear signal in many neurons, likely reflecting ANAs.** **Traceable ANAs.** ENA differentiation and testing for ds-DNA antibodies remained negative. ANCAs, antiphospholipid, and AMAs/SMAs were negative. Rheumatoid factor was negative. Analyses of the complement system showed normal findings for C3, C4, and CH50. **C3d was slightly increased** (9.6 mg/l, reference: < 9 mg/l). Serum IgG and IgA levels normal, **IgM was slightly decreased** (0.34 g/l; reference 0.7–4.0 g/l).
**Cerebrospinal fluid analyses**	Normal white blood cell count (1/µL; reference < 5/µL). **Increased protein concentration (563 mg/L; reference < 450 mg/L).** Normal age-corrected albumin quotient: 8 (age-dependent reference < 9.3 × 10^−3^). No intrahtecal IgG, IgA or IgM synthesis. No CSF specific oligoclonal bands; IgG index not increased (0.53; reference < 0.7). IgG autoantibodies against neuronal cell surface antigens (NMDA-R, AMPA-1/2-R, GABAB-R, DPPX, LGI1, CASPR2) were negative (using fixed cell biochip assays from Euroimmun®). IgG antibodies against the GlyR were negative (cell-based assay; Laboratory Krone, Bad-Salzuflen, Germany). No antibodies against IgLON5 or mGluR5/1 were detected using cell-based assays (Laboratory Krone, Bad Salzuflen, Germany). No IgG autoantibodies against the following intracellular onconeural antigens, amphiphysin, CV2/CRMP5, Ma2/Ta, Ri, Yo, Hu, recoverin, Sox1, Titin, Zic4, DNER/Tr were found using immune blot method (Laboratory Krone, Bad Salzuflen, Germany). Tau/phospho-Tau levels and ß-amyloid quotient were normal. **“Tissue-based assay” using indirect immunofluorescence on unfixed murine brain tissue (Prof. Prüss, Berlin) showed a perinuclear signal in many neurons, likely reflecting ANAs.**
**Cerebral magnetic resonance imaging**	**Combined voxel- and region-based morphometry shows decreased gray matter volumes of the medial frontal lobes (z-scores < −2).** **On visual analysis the midbrain is atrophic**, however the midbrain to pons ratio is with 0.61 in the normal range.
**Spinal magnetic resonance imaging**	No evidence of myelopathy, no evidence of intraspinal contrast-affine lesions.
**Electroencephalo-graphy**.	Visual assessment: No slow wave activity. No epileptic activity. No dysrhythmia. Independent component analysis: Alpha at 10.1 Hz, no abnormal activity.
**[^18^F]fluorodeoxy-glucose positron emission tomography (FDG PET)**	Brain: **Moderate bilateral hypometabolism of the frontal lobes, midbrain and, to a lesser extent, caudate nuclei**. Whole body: No lesions or metabolic changes suspicious of malignancy on whole-body FDG PET/computer tomography.
**[^123^]FPCIT single-photon emission computed tomography (FPCIT SPECT)**	**Severely reduced dopamine transporter availability in both striata, indicating pronounced nigrostriatal degeneration.** The approximated binding potential values were: Caudate nucleus right/left 0.38/0.42 (asymmetry index −10%) and putamen right/left 0.22/0.18 (21%).
